# d-GDM: A mobile diagnostic decision support system for gestational diabetes

**DOI:** 10.20945/2359-3997000000171

**Published:** 2019-08-28

**Authors:** Waldemar Volanski, Ademir Luiz do Prado, Yusra Al-Lahham, Adriana Teleginski, Fabiana Santos Pereira, Dayane Alberton, Fabiane Gomes de Moraes Rego, Glaucio Valdameri, Geraldo Picheth

**Affiliations:** 1 Universidade Federal do Paraná Programa de Pós-graduação em Ciências Farmacêuticas Universidade Federal do Paraná Curitiba PR Brasil Programa de Pós-graduação em Ciências Farmacêuticas, Universidade Federal do Paraná, Curitiba, PR, Brasil; 2 Prefeitura Municipal de Curitiba Prefeitura Municipal de Curitiba Curitiba PR Brasil Prefeitura Municipal de Curitiba, Curitiba, PR, Brasil; 3 Instituto Federal de Educação, Ciência e Tecnologia do Paraná Instituto Federal de Educação, Ciência e Tecnologia do Paraná PR Brasil Instituto Federal de Educação, Ciência e Tecnologia do Paraná, PR, Brasil

**Keywords:** Gestational diabetes, medical informatics, software, decision support, mobile application

## Abstract

**Objective:**

The aim of the study is to describe a portable and convenient software to facilitate the diagnostics of gestational (GDM) and pre-gestational diabetes (PGDM).

**Materials and methods:**

An open source software, d-GDM, was developed in Java. The integrated development environment Android Studio was used as the Android operational system. The software for GDM diagnosis uses the criteria endorsed by the International Association of Diabetes and Pregnancy Study Group, modified by the World Health Organization.

**Results:**

GDM diagnosis criteria is not simple to follow, therefore, errors or inconsistencies in diagnosis are expected and could delay the appropriate treatment. The d-GDM, was developed to assist GDM diagnosis with precision and consistency diagnostic reports. The open source software can be manipulated conveniently. The operator requires information regarding the gestational period and selects the appropriate glycaemic marker options from the menu. During operation, pressing the button “diagnosticar” on the screen will present the diagnosis and information for the follow up. d-GDM is available in Portuguese or English and can be downloaded from the Google PlayStore. A responsive web version of d-GDM is also available. The usefulness and accuracy of d-GDM was verify by field tests involving 22 subjects and 5 mobile phone brands. The approval regards user-friendliness and efficiency were 95% or higher. The GDM diagnosis were 100% correct, in this pilot test. d-GDM is a user-friendly, free software for diagnosis that was developed for mobile devices. It has the potential to contribute and facilitate the diagnosis of gestational diabetes for healthcare professionals.

## INTRODUCTION

Gestational diabetes mellitus (GDM) is the most common complication associated with pregnancy (1,2). Chronic hyperglycemia in pregnancy is associated with several complications in pregnant women and the fetus (3,4).

The definition of gestational diabetes has been changing over time (5). Until recently, a simple definition characterized GDM as “carbohydrate intolerance first diagnosed during gestation” (6,7). Currently, GDM is defined as “a carbohydrate intolerance of variable severity, which begins during current gestation and does not meet the diagnostic criteria for overt diabetes mellitus” (8,9). Therefore, the concept of pre-existing diabetes or pre-gestational diabetes was introduced (10).

Globally, the prevalence of GDM is between 3 and 25% (8). In Brazil, the prevalence of GDM was 18% (11).

**Figure 1:**
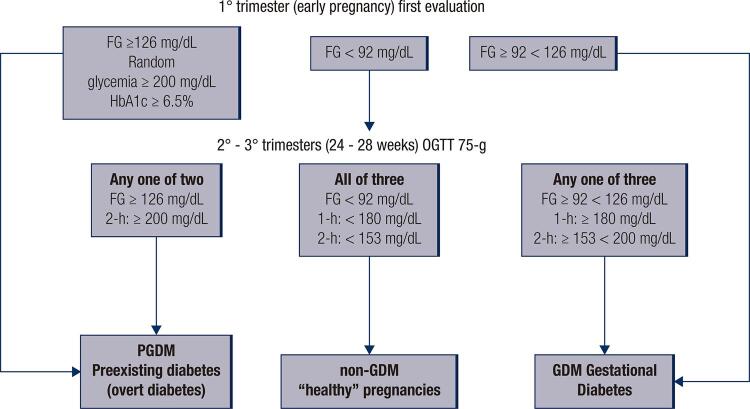
Flowchart for Gestational and Pregestational diagnosis as recommended by IAPDSG-WHO. During the first trimester, the diagnostic criteria for GDM is briefly based on fasting glycemia. Pregestational diabetes (PGDM), also known as preexisting diabetes, overt diabetes, manifest diabetes, or “diabetes in pregnancy” is characterized in this period using the same criteria applied to nonpregnant women (14). All pregnant women, without previous dysglycemia diagnosis, should be tested using the glucose oral tolerance test (OGTT) with a glucose load of 75 g between 24−28 weeks of gestation (2º/3º trimesters). In OGTT only one value above the cut-off is required for GDM or PGDM diagnosis.

GDM diagnosis is based on the blood measurement of glycemia while fasting and/or after an oral glucose load (8,12). Different diagnostic criteria and cut-off values for GDM were proposed (5,13). Figure 1 summarizes the GDM diagnosis as preconized by the International Association of Diabetes and Pregnancy Study Group (IADPSG) with modification by the World Health Organization (WHO) (9).

The described criteria in Figure 1 is based on the findings of the Hyperglycemia and Adverse Pregnancy Outcome (HAPO) study, and the cut-off values proposed by IADPSG (3,14). WHO endorsed the IAPDSG criteria for GDM with minor modifications (9).

The diagnosis of GDM and PGDM (Figure 1) is not simple to follow and requires continuous training of healthcare professionals.

The benefit of information technology (IT) applied to health care is well known (15-17). Several studies have reported the benefits of IT for interventions, improved clinical decision, and improved training and support for healthcare professionals (18-20).

The aim of the study is to describe a portable and convenient software to facilitate the diagnosis of GDM and PGDM.

## MATERIALS AND METHODS

The Federal University of Parana’s Ethics Committee approved this study (CAAE: 39460414.0.0000.0102 and CAAE: 68027317.7.0000.0102).

### Software development

The software was built using the variables and criteria suggested by WHO. These variables are as follows: fasting glycemia, random glycemia, and glycated hemoglobin (HbA1c) in the first trimester (first evaluation). Subsequently, fasting glycemia and glycemia after 1 hr and 2 hr, after an oral glucose tolerance test (OGTT) with a glucose load of 75 g during a period of 24-28 weeks of gestation (as described in Figure 1).

All informatics tolls that were applied are open-source. *Java* was used for development. d-GDM was developed for the *Android* Operating Systems using the Android Studio (https://developer.android.com/studio/) integrated development environment (IDE), in which *IntelliJ IDEA* from JetBrains is applied.

d-GDM was developed considering the principles of human-computer interaction (HCI). HCI is a field that involves the interaction between people (users) and computational devices, developing methods and tools for the design, creation, and implementation and maintenance of computational systems suitable for human use (21). HCI seeks to minimize the barrier between the human user and the computer system by offering user-friendly interfaces that can be used with the least effort (22).

**Figure 2:**
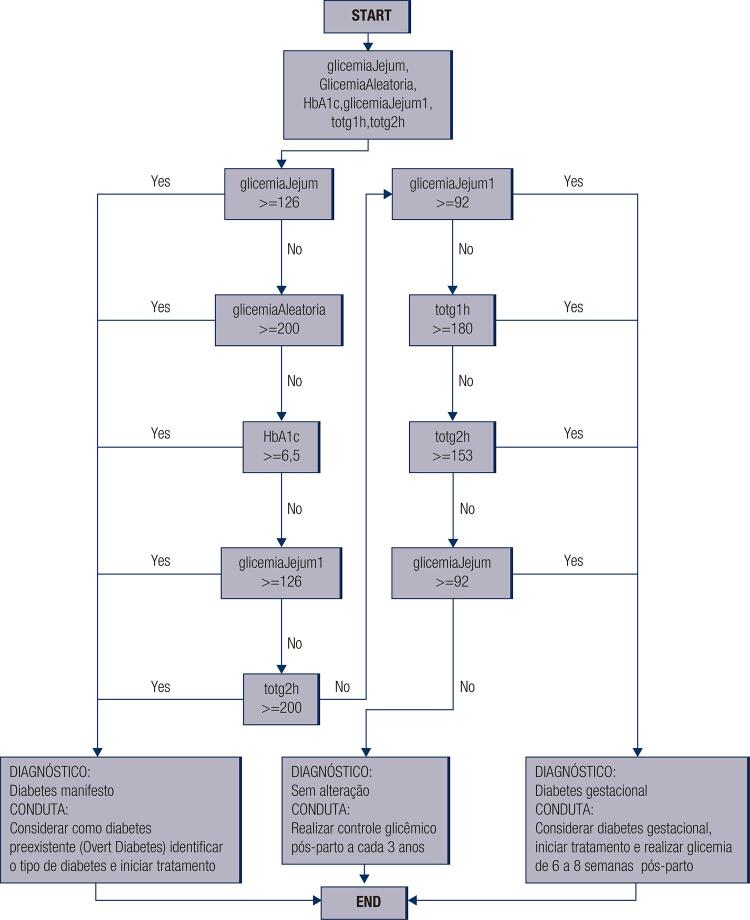
d-GDM flowchart of the programing logic. The main variables used in the program were fasting glycemia (glicemiaJejum), random glycemia (GlicemiaAleatoria), glycated hemoglobin A1c (HbA1c), fasting glycemia during OGTT (glicemiaJejum1), glycemia 1-hour after 75-g oral glucose load (totg1h), and glycemia 2-hour (totg2h).

The flowchart of the programming logic is shown in Figure 2.

The programming logic is described briefly below.

if (btn2.isChecked() || btn4.isChecked() || btn6.isChecked() || btn9.isChecked() || btn14.isChecked()) {diagnostico = “Diabetes Manifesto. Considerar como diabetes preexistente, identificar tipo de diabetes e Iniciar tratamento.”} else if (btn15.isChecked() || btn8.isChecked() || btn11.isChecked() || btn13.isChecked()) {diagnostico = “Diabetes Gestacional. Realizar glicemia de 6 a 8 semanas pós-parto.”; }else {diagnostico = “Sem diabetes. Realizar controle Glicêmico pós-parto a cada 3 anos.”;}

The above script represents the core of the program. “btn” is a button associated with the variable defined at the front-end. All other are associated with programming syntax.

The Android application program was compilated to into Android application package (.apk) and transferred to a smartphone.

The d-GDM apk was uploaded to the Google PlayStore (https://play.google.com/store), where it can be downloaded. An English version is automatically installed when the Operating System is different from Portuguese language.

d-GDM software requires a hard disk space of 2.65 Mb, and is ISO 9126-1 compliant, which preconize functionality, reliability, usability, efficiency, maintainability, and portability.

The open-source d-GDM software, as described above, was registered in the Brazilian INPI – *Instituto Nacional da Propriedade Industrial* (National Institute of Industrial Property) under the number BR512018001275-2, linked to the Federal University of Parana, Brazil.

A responsive web version (adaptable and flexible to all computer screens) was developed and can be accessed at http://www.cpdm.ufpr.br/dg/.

## RESULTS

**Figure 3:**
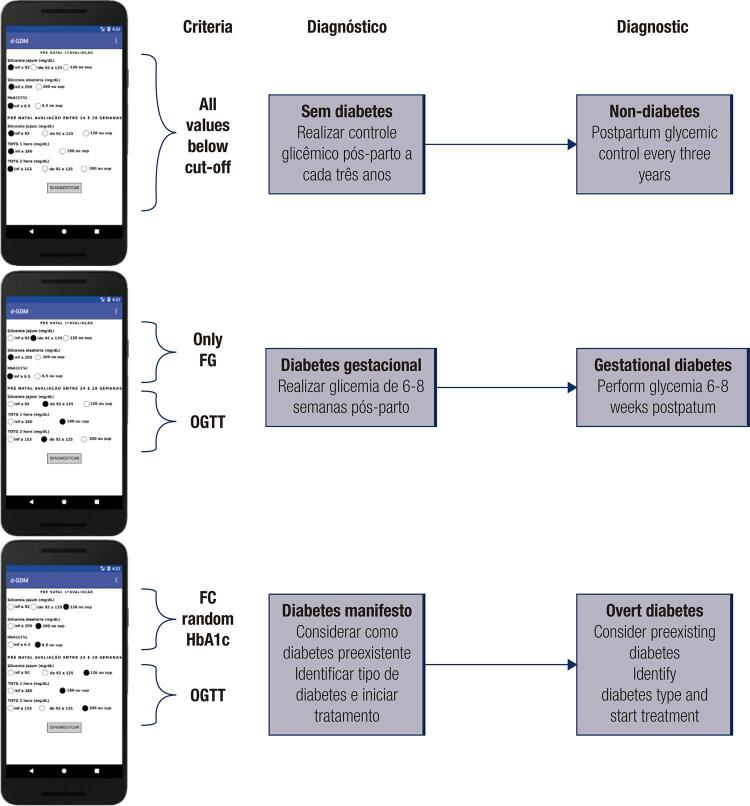
d-GDM software major diagnostics templates.

The major characteristics of the d-GDM software are shown in Figure 3.

In Figure 3, a pregnant woman is considered as an example. In the first screenshot, all variables radio buttons were below the cut-off for diabetes. Tapping the “diagnosticar” button shows the diagnostic of no-diabetes, in Portuguese, in this version. In the second screenshot, the user has two options. Selecting the variables for the 1º trimester or for the 2º/3º trimesters (24−28 weeks of gestation) will cause the appropriate diagnostic to be displayed, such as GDM in the example. In the third screenshot, all radio buttons were in the highest cut-off and the “diagnosticar” bottom will display the diagnostics for preexisting diabetes (overt diabetes).

The open-source software shows a screenshot in Portuguese or English. It is easy and simple to use. The user requires information regarding the gestational period and select the appropriate glycemic marker options. Pressing the button “*diagnosticar*” (or “submit”, English version) in this scenario will result in the diagnosis and information regarding the follow up to be presented on the screen.

The usefulness and accuracy application of d-GDM was verified by field tests involving graduate students (n = 22) using five different mobile phone brands. Questions on user-friendliness, language and efficiency showed scores of 95% or higher associated with highest approval of d-GDM (data not shown). Suggestions from the users regarding the interface layout of the app were incorporated in d-GDM final version.

To verify the d-GDM accuracy, the group of post-graduate students received a 30 minutes training on GDM diagnostic with the flowchart described in Figure 1.

Following, the group received a spreadsheet (Microsoft Excel) with 50 results of fasting glycemia and OGTT, randomly containing 25% of positive results for GDM, confirmed by two senior researchers; they mark (Yes or No) for GDM. This procedure mimics the “usual diagnostic” process.

The same spreadsheet was then shuffled, and the group performed the diagnosis with the d-GDM app, after a 5 minutes training, blinded for the first results.

The study group presented a correct diagnosis in 94% with the “usual” procedure and 100% using the d-GDM app. Regardless of the small sample size, this preliminary result for accuracy was considered consistent and satisfactory for d-GDM.

## DISCUSSION

The decision to develop a software program to assist the diagnosis of gestational diabetes was motivated by difficulties encountered by healthcare professionals.

The evolution in the definition of GDM and the different options for cut-off values of glycemic biomarkers and glucose loads could be relevant factors affecting diagnosis consistency.

Therefore, errors in diagnosis are expected and could delay the appropriate treatment. The d-GDM, was developed to assist GDM diagnosis with precision and consistency diagnostic reports.

In addition, rapid and continuous changes in the guidelines for the diagnosis of GDM have been observed, requiring constant updating of the professionals and favouring the use of non-standard criteria.

We developed a free application for mobile devices. This application is an intuitive and simple way to diagnose GDM.

The principles of good interaction between the health professionals and the d-GDM application were considered during development. A simplified screen, without the need to advance to subsequent screenshot was developed, thereby reducing unnecessary visualizations. This development yields desirable characteristics, according to the concepts of HCI (21,22).

The software was built with a radio button, also known as an “option button”, which is graphical element that allows a user to select a single item from a predefined list of options. This prevents typographic errors associated with concentrations of biomarkers (Figure 3). The program operator selects simple choices and obtains an immediate response. The d-GDM application interface is designed, based on the models provided for the Android-based platform.

A new criterion for the diagnosis of GDM that may be considered in the future can easily be incorporated into d-GDM. The software structure of d-GDM presents a consolidated and flexible logic.

In Brazil, the number of mobiles in operation exceeds the number of inhabitants (23). Health professionals have been expanding the use of multiple mobile-based applications for diagnostic assistance (24,25).

Different technological processes, involving *in silico* analyses such as the use of artificial intelligence, associated with diabetes, are available and expand rapidly (26). These new technologies, in diabetes, are focused on processes of prevention, monitoring and research, for what has been described as “digital diabetes” (27).

The use of softwares available in mobile phones has been associated with improved glycemic control and self-management in diabetes care in type 2 diabetes, confirming the effectiveness of this technology (28).

Gestational diabetes softwares and apps, identified on the main download platforms, are primarily targeted at pregnant women, addressing glycemic control, weight management, lifestyle modifications, drug control and monitoring, and complications (29).

Different digital mobile health medicine (mHealth) software for gestational diabetes have been associated with reduced risk for the pregnant woman and the fetus (27,30). d-GDM is a software that fits into the emerging mHealth context.

The d-GDM was developed with the interactive process described by Garnweidner and cols. in 2015 (31). Briefly, a multidisciplinary health group, associated with computer and bioinformatics experts, developed the software prototype, which was improved by users.

Healthcare professionals are exposed to constant stress. After long hours of work, misunderstandings and errors are expected, especially in complex diagnoses such as GDM and PGDM.

Therefore, errors in diagnosis are expected and could delay the appropriate treatment. The d-GDM, was developed to assist GDM diagnosis with precise and consistency diagnostic reports.

In addition, rapid and continuous changes in the guidelines for the diagnosis of GDM have been observed, requiring constant updating of the professionals and favouring the use of non-standard criteria.

d-GDM was developed for operation in an Android Operating System, which is the most popular in the world. This makes the system accessible to the largest number of mobile equipment in use. To our knowledge, d-GDM is the first software to address GDM and PGDM diagnosis to mobile devices.

Additionally, any computer can access the d-GDM web version. This option was developed for users who do not use the Android operating system. In the web version, more information such as the logic of the application and the diagnostic criteria, as well as the bibliographic references, are provided. The website also enables better communication between users and the developers of d-GDM, as well as the dissemination of the projects developed in gestational diabetes research.

In this context, the proposed d-GDM software can provide healthcare professionals with a free tool that facilitates the diagnosis of gestational diabetes, which is an issue of significant and increasing prevalence in Brazil and in the world.

To our knowledge d-GDM was the first-of-its-kind that is able to address the GDM diagnosis.

In conclusion, in this study, we developed and described d-GDM, which is a free software for GDM diagnosis. d-GDM is user-friendly and has the potential to assist healthcare professionals in the diagnosis of gestational diabetes.

## References

[1] Coton SJ, Nazareth I, Petersen I. A cohort study of trends in the prevalence of pregestational diabetes in pregnancy recorded in UK general practice between 1995 and 2012. BMJ Open. 2016;6(1):e009494. 10.1136/bmjopen-2015-009494 PMC4735208 26810997

[2] McIntyre HD. Diagnosing gestational diabetes mellitus: rationed or rationally related to risk? Diabetes Care. 2013;36(10):2879-80. 10.2337/dc13-1250 PMC3781537 24065840

[3] Metzger BE, Lowe LP, Dyer AR, Trimble ER, Chaovarindr U, Coustan DR, et al. Hyperglycemia and adverse pregnancy outcomes. N Engl J Med. 2008;358(19):1991-2002. 10.1056/NEJMoa0707943 18463375

[4] Lowe WL Jr, Scholtens DM, Lowe LP, Kuang A, Nodzenski M, Talbot O, et al. Association of Gestational Diabetes With Maternal Disorders of Glucose Metabolism and Childhood Adiposity. JAMA. 2018;320(10):1005-16. 10.1001/jama.2018.11628 PMC6143108 30208453

[5] Wexler DJ, Powe CE, Barbour LA, Buchanan T, Coustan DR, Corcoy R, et al. Research Gaps in Gestational Diabetes Mellitus: Executive Summary of a National Institute of Diabetes and Digestive and Kidney Diseases Workshop. Obstet Gynecol. 2018;132(2):496-505. 10.1097/AOG.0000000000002726 PMC6124493 29995731

[6] The-Expert-Committee-On-The-Diagnosis-And-Classification-Of-Diabetes-Mellitus. Report of the Expert Committee on the Diagnosis and Classification of Diabetes Mellitus. Diabetes Care. 1997;20(7):1183-97. 10.2337/diacare.20.7.1183 9203460

[7] SBD. Diretrizes da Sociedade Brasileira de Diabetes 2014-2015. Sociedade Brasileira de Diabetes. 2015.

[8] de Oliveira JEP, Montenegro Junior RM, Vencio S (orgs.). Diretrizes da Sociedade Brasileira de Diabetes 2017-2018. São Paulo: Editora Clannad, 2017.

[9] WHO. Diagnostic criteria and classification of hyperglycaemia first detected in pregnancy: a World Health Organization Guideline. Diabetes Res Clin Pract. 2014;103(3):341-63. 10.1016/j.diabres.2013.10.012 24847517

[10] International Association of Diabetes in Pregnancy Study Group Working Group on Outcome D, Feig DS, Corcoy R, Jensen DM, Kautzky-Willer A, Nolan CJ, et al. Diabetes in pregnancy outcomes: a systematic review and proposed codification of definitions. Diabetes Metab Res Rev. 2015;31(7):680-90. 10.1002/dmrr.2640 25663190

[11] Trujillo J, Vigo A, Duncan BB, Falavigna M, Wendland EM, Campos MA, et al. Impact of the International Association of Diabetes and Pregnancy Study Groups criteria for gestational diabetes. Diabetes Res Clin Pract. 2015;108(2):288-95. 10.1016/j.diabres.2015.02.007 25765668

[12] ADA. Standards of Medical Care in Diabetes – 2018. Diabetes Care. 2018;41(1):172.

[13] Agarwal MM. Gestational diabetes mellitus: Screening with fasting plasma glucose. World J Diabetes. 2016;7(14):279-89. 10.4239/wjd.v7.i14.279 PMC4958688 27525055

[14] International Association of Diabetes and Pregnancy Study Groups Consensus Panel, Metzger BE, Gabbe SG, Persson B, Buchanan TA, Catalano PA, Damm P, et al. International association of diabetes and pregnancy study groups recommendations on the diagnosis and classification of hyperglycemia in pregnancy. Diabetes Care. 2010;33(3):676-82. 10.2337/dc09-1848 PMC2827530 20190296

[15] Haux R. Health information systems – past, present, future. Int J Med Inform. 2006;75(3-4):268-81. 10.1016/j.ijmedinf.2005.08.002 16169771

[16] Kvedar J, Coye MJ, Everett W. Connected health: a review of technologies and strategies to improve patient care with telemedicine and telehealth. Health Affairs. 2014;33(2):194-9. 10.1377/hlthaff.2013.0992 24493760

[17] Pritchard DE, Moeckel F, Villa MS, Housman LT, McCarty CA, McLeod HL. Strategies for integrating personalized medicine into healthcare practice. Per Med. 2017;14(2):141-52. 10.2217/pme-2016-0064 29754553

[18] Garg AX, Adhikari NK, McDonald H, Rosas-Arellano MP, Devereaux PJ, Beyene J, et al. Effects of computerized clinical decision support systems on practitioner performance and patient outcomes: a systematic review. JAMA. 2005;293(10):1223-38. 10.1001/jama.293.10.1223 15755945

[19] Chaudhry B, Wang J, Wu S, Maglione M, Mojica W, Roth E, et al. Systematic review: impact of health information technology on quality, efficiency, and costs of medical care. Ann Intern Med. 2006;144(10):742-52. 10.7326/0003-4819-144-10-200605160-00125 16702590

[20] Caburnay CA, Graff K, Harris JK, McQueen A, Smith M, Fairchild M, et al. Evaluating diabetes mobile applications for health literate designs and functionality, 2014. Prev Chronic Dis. 2015;12:E61. 10.5888/pcd12.140433 PMC4436041 25950568

[21] Arsand E, Froisland DH, Skrovseth SO, Chomutare T, Tatara N, Hartvigsen G, et al. Mobile health applications to assist patients with diabetes: lessons learned and design implications. J Diabetes Sci Technol. 2012;6(5):1197-206. 10.1177/193229681200600525 PMC3570855 23063047

[22] Ventola CL. Mobile devices and apps for health care professionals: uses and benefits. P T. 2014;39(5):356-64. PMC4029126 24883008

[23] Brasil. Ministério das Comunicações. Agência Nacional de Telecomunicações. Telefonia Móvel. Available from: http://www.anatel.gov.br/dados/acessos-telefonia-movel2015. Access on: Jul. 22 2019.

[24] El-Gayar O, Timsina P, Nawar N, Eid W. Mobile applications for diabetes self-management: status and potential. J Diabetes Sci Technol. 2013;7(1):247-62. 10.1177/193229681300700130 PMC3692239 23439183

[25] García-Sánchez P, González J, Mora AM, Prieto A. Deploying intelligent e-health services in a mobile gateway. Expert Syst Appl. 2013;40(4):9.

[26] Unnikrishnan R, Sharma N, Mohan V, Ranjani H. Technology in the management of Diabetes mellitus. J Diabetol. 2018;9:3-11.

[27] Fagherazzi G, Ravaud P. Digital diabetes: Perspectives for diabetes prevention, management and research. Diabetes Metab. 2018 Sep 19. pii: S1262-3636(18)30171-X. 10.1016/j.diabet.2018.08.012 30243616

[28] Liang X, Wang Q, Yang X, Cao J, Chen J, Mo X, et al. Effect of mobile phone intervention for diabetes on glycaemic control: a meta-analysis. Diabet Med. 2011;28(4):455-63. 10.1111/j.1464-5491.2010.03180.x 21392066

[29] Chen Q, Carbone ET. Functionality, implementation, impact, and the role of health literacy in mobile phone apps for gestational diabetes: scoping review. JMIR Diabetes. 2017;2(2):e25. 10.2196/diabetes.8045 PMC6238859 30291088

[30] Guo H, Zhang Y, Li P, Zhou P, Chen LM, Li SY. Evaluating the effects of mobile health intervention on weight management, glycemic control and pregnancy outcomes in patients with gestational diabetes mellitus. J Endocrinol Invest. J Endocrinol Invest. 2019 Jun;42(6):709-14. 10.1007/s40618-018-0975-0 30406378

[31] Garnweidner-Holme LM, Borgen I, Garitano I, Noll J, Lukasse M. Designing and developing a mobile smartphone application for women with gestational diabetes mellitus followed-up at diabetes outpatient clinics in Norway. Healthcare (Basel). 2015;3(2):310-23. 10.3390/healthcare3020310 PMC4939538 27417764

